# 肺叶与亚肺叶切除治疗60岁以上Ia期小细胞肺癌的预后比较分析

**DOI:** 10.3779/j.issn.1009-3419.2018.01.02

**Published:** 2018-01-20

**Authors:** 海康 曾, 洋 刘, 晓君 夏, 谨 李, 建行 何

**Affiliations:** 1 510120 广州，广州医科大学附属第一医院胸外科 Department of Thoracic Surgery, the First Affiliated Hospital of Guangzhou Medical University, Guangzhou 510120, China; 2 510120 广州，呼吸疾病国家重点实验室 State Key Laboratory of Respiratory Disease, the First Affiliated Hospital of Guangzhou Medical University, Guangzhou 510120, China

**Keywords:** 小细胞肺癌, 亚肺叶切除, 肺叶切除, 放化疗, 预后, SCLC, Sub-lobectomy, Lobectomy, Radiochemotherapy, Prognosis

## Abstract

**背景与目的:**

目前，肺叶切除与亚肺叶切除治疗Ia期小细胞肺癌（small cell lung cancer, SCLC）的预后比较鲜有报道。本研究通过对年龄≥60岁的T1N0M0（≤3 cm）SCLC进行回顾性研究，旨在探索肺叶切除与亚肺叶切除治疗Ia期SCLC预后的对比分析。

**方法:**

纳入“监测，流行病学和结果数据库”（Surveillance, Epidemiology and End Results database, SEER）在1992年-2010年间经病理诊断为Ia期SCLC患者共515例，数据使用*Kaplan-Meier*（*Log-rank*检验）和*Cox*模型进行比较统计分析。

**结果:**

肺叶切除组（*n*=110）、亚肺叶切除组（*n*=57）、和非手术治疗组（*n*=348）的中位生存期分别为45个月、23个月和16个月；该三组相应的5年总生存期（OS）分别为44%、30%和14%（Lob *vs* Sub, χ^2^=4.851, *P*=0.028; Sub *vs* non-surgical, χ^2^=6.529, *P*=0.011）。SCLC有、无淋巴结采样/清扫患者的预后无显著差异（*P*=0.107）；肺叶切除+放化疗组（Lob+CR, *n*=59）的5年OS为50%。*Cox*分析证实，手术（肺叶与亚肺叶切除术）方式为独立预后预测因素之一。

**结论:**

年龄≥60岁的Ia期SCLC患者，我们推荐解剖性肺叶切除联合辅助放化疗治疗。

## Abstract (en)

**Background and objective:**

Currently, the prognosis of lobectomy and sub-lobectomy for the treatment of stage Ia small cell lung cancer (SCLC) is rarely reported. We retrospectively studied T1N0M0 (≤3 cm) SCLC patients aged ≥60 years, aiming to comparatively analyze the prognosis of lobectomy and sub-lobectomy in treating patients with Ia SCLC.

**Methods:**

Patients with stage Ia SCLC diagnosed by pathologic between 1992 and 2010 were selected from the "Surveillance, Epidemiology and End Results database"(SEER). Outcome data were compared using *Kaplan-Meier* (*Log-rank* test) and *Cox* model multivariate analysis.

**Results:**

We identified 515 patients. Median overall survival (OS) of the lobectomy (*n*=110), sublobar resection (*n*=57) and non-surgical (*n*=348) cohort were 45, 23 and 16 months, respectively. The corresponding 5-year OS of the three groups were 44%, 30%, and 14%, respectively. No significant difference in the prognosis of patients with or without lymph node examination/ dissection (*P*=0.107) and the 5-year OS of patients underwent lobectomy with chemoradiation was 50%. *Cox* multivariable analysis showed that operation treatment, including lobectomy and sublobectomy, was one of the independent factors associated with the prognosis of early SCLC patients, and patients undergo lobectomy shows a better OS compared with sublobar resection (Lob *vs* Sub, HR=0.645; 95%CI: 0.433-0.961, *P*=0.031).

**Conclusion:**

For age ≥60 years T1N0M0 (≤3 cm) SCLC patients, we recommend anatomical lobectomy combined with adjuvant chemoradiation.

小细胞肺癌（small cell lung cancer, SCLC）是肺癌中一类特殊的恶性实体瘤，约占肺癌总数的10%-15%，发病与长期吸烟有关^[1]^。SCLC的特点是侵袭性强、增长迅速、早期易转移、具有癌旁内分泌特性，对放疗、化疗治疗较敏感^[1, 2]^，因此，目前的治疗多以化疗或放疗为主^[3]^。近年来，研究显示早期SCLC患者如病变仅局限于肺实质内，最初治疗方案考虑以手术治疗为主^[4, 5]^，最新的美国国立综合癌症网络（National Comprehensive Cancer Network, NCCN）指南表明，对于明确无淋巴结转移的Ⅰ期（T1-2N0）SCLC患者，推荐行手术治疗^[6]^。

目前指南已强烈推荐，电视辅助胸腔镜（video assisted thoracic surgery, VATS）微创手术治疗肺癌，标准术式为肺叶切除术。对于早期小肺癌（≤3 cm），亚肺叶切除（楔形/肺段）术式可在完整切除肿瘤的基础上、最大限度地保护了患者肺功能，现已较普遍应用于早期肺癌的外科治疗^[7, 8]^。已有多项研究证实，亚肺叶切除术式治疗对各年龄段、早期非小细胞肺癌（non-small cell lung cancer, NSCLC）的生存预后结果中，取得了与肺叶切除治疗类似的术后预后^[9-13]^。

有术者考虑到亚肺叶（楔形/肺段）切除治疗的优势和SCLC快速生长、对化放疗敏感等特性，认为亚肺叶切除或可作为SCLC的姑息性手术治疗方案之一，尤其是针对心肺功能衰退的老年SCLC患者。然而，目前关于亚肺叶切除治疗IaA期SCLC、以及与肺叶切除的对比研究及预后报道较少，早期SCLC的手术治疗方式（肺叶/亚肺叶切除）和选择仅为NCCN专家组的倾向意见，缺少有力的循证医学证据支持。

鉴于此，本研究通过选择年龄≥60岁的T1N0M0（≤3 cm）SCLC患者，进行回顾性研究和统计分析，旨在探索肺叶切除与亚肺叶切除治疗早期SCLC生存预后的对比，以及影响术后预后的相关临床病理因素。

## 1 资料和方法

### 1.1 资料

通过美国国立癌症研究所“监测、流行病学和结果数据库（Surveillance, Epidemiology and End Results database, SEER）”数据库，使用SEER^*^Stat8.3.4软件检索该数据库，获取SCLC的临床病理特征资料及相关预后信息。本研究最终纳入1992年1月-2010年12月，共515例SCLC患者。纳入标准：（1）病理证实T1N0 SCLC（≤3 cm）；（2）年龄≥60岁；（3）存在一个原发性恶性病变[按照国际肺癌研究协会（International Association for the Study of Lung Cancer, IASLC）第7版TNM分期标准，pT1N0M0]。

根据SEER数据库，获取了以下信息：患者基本信息（年龄、性别、种族）、肿瘤相关特征（大小及位置）、淋巴结检查、放化疗、外科手术治疗及预后情况。总队列中，将患者分为肺叶切除（Lob）、亚肺叶切除（Sub）和非手术队列（Non-surgical）。在手术（肺叶+亚肺叶切除）队列中，首先根据是否行淋巴结采样/清扫分为2个亚组（是：*n*=99；否：*n*=43）；然后根据术后有无放化疗分为4个亚组：肺叶切除加放化疗组（Lob+CR, *n*=59）、单独行肺叶切除组（Lob, *n*=51）、亚肺叶切除加放化疗组（Sub+CR, *n*=35）、单独行亚肺叶切除组（Sub, *n*=22）。

### 1.2 研究结果及随访

本研究的终止日期为2013年12月31日，总生存期（overall survival, OS）定义为从确诊SCLC开始，至因任何原因引起死亡的时间间隔；失访或研究终止日期仍存活定义为删失。肺癌特异性生存期（lung cancer specific survival, LCSS）定义为确诊SCLC至肺癌致死的时间间隔；失访或其他死亡原因致死或研究终止日期仍存活则定义为删失。该研究总队列的平均随访时间（median follow-up time, MFT）为36（范围0-120）个月。

### 1.3 统计学方法

计量资料用均数±标准差（Mean±SD）表示，计数资料用百分比（%）表示。*Kaplan-Meier*法（*Log-rank*检验）比较生存曲线及统计学差异。用*Kaplan-Meier*法单因素分析得出有意义的临床病理因素纳入*Cox*回归模型进行多因素分析。所有数据均采用SPSS 20.0软件进行统计学分析。生存曲线使用GraphPad Prism 5.01软件作图。检验水准：*P* < 0.05有统计学意义。

## 2 结果

### 2.1 一般结果

本研究纳入1992年1月-2010年12月，共515例≥60岁T1N0M0 SCLC。其中167例（33%）接受手术切除（94例接受术后化疗、22例接受术后放疗、73例仅手术治疗）；348例（68%）接受非手术治疗。整个队列的平均年龄为72岁（60岁-95岁），平均肿瘤大小为2.1（范围0.4 cm-3 cm）cm，平均随访时间为36（范围1个月-120个月）个月。在肺叶切除组（110例）中，78例（71%）患者接受了淋巴结采样及病理检查；亚肺叶切除组中（57例）中，21例（37%）患者接受了淋巴结采样及病理检查。亚肺叶切除手术方式为:楔形切除23例、部分/楔形/节段切除27例（1992年-1997年）、肺段切除4例（表 1）。

**1:** 515例Ia期SCLC患者的临床特征[*n*(%)] Clinical characteristics of 515 patients with stage Ia SCLC[*n*(%)]

Variable	No (%) of patients			*P*
	Lob (*n*=110)	Sub (*n*=57)	Non-surgical (*n*=348)	
Age (Mean±SD, yr)	70±6	74±6	72±7	0.002
Age group (yr)				0.009
60-74	84 (76.4)	33 (57.9)	213 (61.2)	
75	26 (23.6)	24 (42.1)	135 (38.8)	
Gender				0.600
Male	55 (50.0)	24 (42.1)	160 (46.0)	
Female	55 (50.0)	33 (57.9)	188 (54.0)	
Race				0.120
White	97 (88. 2)	54 (94.7)	296 (85.1)	
Black/other	5/8 (11.8)	1/2 (5.3)	33/19 (14.9)	
Location				0.019
Upper	63 (57.3)	31 (54.4)	216 (62.1)	
Middle	15 (13.6)	3 (5.3)	17 (4.9)	
Lower	32 (29.1)	23 (40.3)	115 (33.0)	
Tumor size (cm)				< 0.001
≤2	62 (56.4)	46 (80.7)	162 (46.6)	
2-3	48 (43.6)	11 (19.3)	186 (53.4)	
ELN count				< 0.001
None/unknown	11/22 (29.7)	32/4 (63.2)	334/14 (97.5)	
≥1	78 (70.3)	21 (36.8)	9 (2.5)	
Radiation				< 0.001
No/unknown	96/1 (88.2)	47/1 (84.2)	153/9 (46.6)	
Yes	13 (11.8)	9 (15.8)	186 (53.4)	
Chemotherapy				0.003
No/unknown	51 (46.4)	22 (38.6)	102 (29.3)	
Yes	59 (53.6)	35 (61.4)	246 (70.7)	
MFT (mo)	58	48	26	< 0.001
Sub: sublobar resection; Lob: lobectomy; SD: standard deviation; ELN: examined lymph node; MFT: mean follow-up time.				

### 2.2 总队列的OS及LCSS

本研究中肺叶切除组、亚肺叶切除组、和非手术治疗组的中位OS分别为45个月、23个月和16个月，该三组相应的5年OS分别为44%、30%和14%（Lob *vs* Sub，χ^2^=4.851，*P*=0.028；Sub *vs* non-surgical，χ^2^=6.529，*P*=0.011；图 1A）。中位LCSS在肺叶切除组、亚肺叶切除组、和非手术治疗组中分别为65个月、29个月和18个月，该三组相应的5年LCSS分别为55%、41%和21%（Lob *vs* Sub，χ^2^=2.694，*P*=0.101；Sub *vs* non-surgical，χ^2^=8.617，*P*=0.003；图 1B）。

**1:**
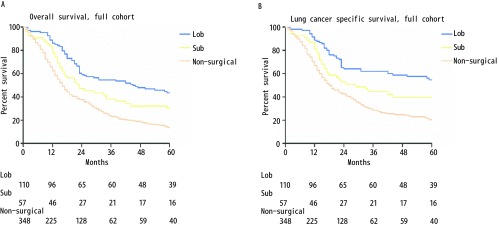
Ia期SCLC患者肺叶切除组、亚肺叶切除组及非手术组的生存曲线. A：OS曲线（Lob *vs* Sub, *χ*^2^=4.851, *P*=0.028; Sub *vs* non-surgical, *χ*^2^=6.529, *P*=0.011）；B：LCSS曲线（Lob vs Sub, *χ*^2^=2.694, *P*=0.101; Sub *vs* non-surgical, *χ*^2^=8.617, *P*=0.003）。 *Kaplan-Meier* survival curves between stage Ia SCLC patients with Lob, Sub and non-surgical cohort. A: OS curve (Lob *vs* Sub, *χ*^2^=4.851, *P*=0.028; Sub *vs* non-surgical, *χ*^2^=6.529, *P*=0.011); B: LCSS curve (Lob *vs* Sub, *χ*^2^=2.694, *P*=0.101; Sub *vs* non-surgical, *χ*^2^=8.617, *P*=0.003).

### 2.3 手术队列的OS和LCSS

在淋巴结采样/清扫检查精确计数的手术队列中（*n*=142），有淋巴结检查（*n*=99）和无淋巴结检查（*n*=43）的患者中位OS分别为42个月和23个月，其相应的中位LCSS分别为64个月和24个月。有无淋巴结检查患者的5年OS分别为42%和31%（有*vs*无，χ^2^=2.600，*P*=0.107；图 2A），相应的5年LCSS分别为52%和41%（有*vs*无，χ^2^=0.275，*P*=0.600；图 2B）。

**2:**
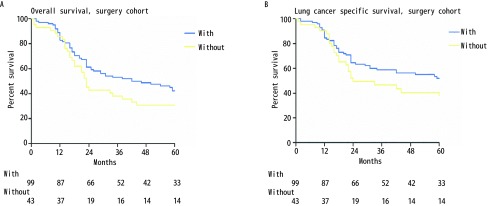
手术队列中淋巴结检查组与对照组的生存曲线。A：OS曲线（有*vs*无，*χ*^2^=2.600，*P*=0.107）；B：LCSS曲线（有*vs*无, *χ*^2^=0.275, *P*=0.600）。 *Kaplan-Meier* survival curves between stage Ia SCLC patients with and without ELN in the surgery cohort. A: OS curve (with *vs* without, *χ*^2^=2.600, *P*=0.107); B: LCSS curve (with *vs* without, *χ*^2^=0.275, *P*=0.600).

### 2.4 术后联合放化疗（CR）比较

总共167例患者被分为以下几组：肺叶切除+放化疗组（Lob+CR, *n*=59）、单独肺叶切除组（Lob, *n*=51）、亚肺叶切除+放化疗组（Sub+CR, *n*=35）、单独亚肺叶切除组（Sub, *n*=22）。该四组的中位OS分别为未定义、29个月、25个月和19.5个月；该四组5年OS分别为：50%、36%、37%和17%（Lob+CR *vs* Lob，χ^2^=4.210，*P*=0.040；Lob *vs* Sub+CR，χ^2^=0.094，*P*=0.759；Sub+CR *vs* Sub，χ^2^=4.091，*P*=0.043；图 3A）。中位LCSS在肺叶切除+放化疗组、单独肺叶切除组、亚肺叶切除+放化疗组及单独亚肺叶切除组中分别为122个月、62个月、111个月和19.5个月；四组5年LCSS分别为57%、52%、50%和20%（Lob + CR *vs* Lob，χ^2^=2.674，*P*=0.102；Lob *vs* Sub+CR，χ^2^=0.466，*P*=0.495；Sub+CR *vs* Sub，χ^2^=4.936，*P*=0.026；图 3B）。

**3:**
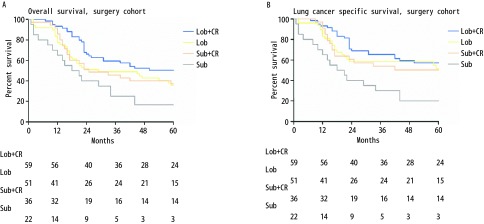
Ia期SCLC患者肺叶切除+放化疗组、单独肺叶切除组、亚肺叶切除+放化疗组及单独亚肺叶切除组的生存曲线。A：OS曲线（Lob+CR *vs* Lob, *χ*^2^=4.210, *P*=0.040; Lob *vs* Sub+CR, *χ*^2^=0.094, *P*=0.759; Sub+CR *vs* Sub, *χ*^2^=4.091, *P*=0.043）；B: LCSS曲线（Lob+CR *vs* Lob, *χ*^2^=2.674, *P*=0.102; Lob *vs* Sub+CR, *χ*^2^=0.466, *P*=0.495; Sub+CR *vs* Sub, *χ*^2^=4.936, *P*=0.02）。 *Kaplan-Meier* survival curves between stage Ia SCLC patients with Lob+CR, Lob, Sub+CR and Sub cohort. A: OS curve (Lob+CR *vs* Lob, *χ*^2^=4.210, *P*=0.040; Lob *vs* Sub+CR, *χ*^2^=0.094, *P*=0.759; Sub+CR *vs* Sub, *χ*^2^=4.091, *P*=0.043); B: LCSS curve (Lob+CR *vs* Lob, *χ*^2^=2.674, *P*=0.102; Lob *vs* Sub+CR, *χ*^2^=0.466, *P*=0.495; Sub+CR *vs* Sub, *χ*^2^=4.936, *P*=0.02).

### 2.5 单因素和多因素统计分析

通过*Kaplan-Meier*法单因素分析显示（表 2）：有意义的临床病理因素为年龄、肿瘤大小、淋巴结检查计数、是否手术、放疗、化疗（*P* < 0.05）。*Cox*多因素分析表明（表 3）：淋巴结检查、手术（肺叶切除/亚肺叶切除）、放化疗是独立的预测预后因素。与亚叶切除术相比，肺叶切除术可改善患者的OS（Lob *vs* Sub, HR=0.645, 95%CI: 0.433-0.961, *P*=0.031），但对LCSS并无明显差异（Lob *vs* Sub, HR=0.703, 95%CI: 0.459-1.078, *P*=0.106）。

**2:** Ia期SCLC患者总生存和肺癌特异性生存的单变量分析 Univariate analysis of overall survival and lung cancer specific survival for Ia SCLC

Variable	Univariate analysis				
	Overall survival			Lung cancer specific survival	
	*χ*^2^	*P*		*χ*^2^	*P*
Age (yr)	13.961	< 0.001		13.473	< 0.001
60-74/75					
Gender	1.762	0.184		0.790	0.374
Male/Female					
Race	2.012	0.147		0.935	0.333
White/black/other					
Location	2.275	0.321		3.972	0.137
Upper/Middle/Lower					
Tumor size (cm)	0.459	0.498		4.824	0.028
≤2/2-3					
ELN count	27.637	< 0.001		22.570	< 0.001
None/≥1					
Surgical Procedure	42.777	< 0.001		42.934	< 0.001
Lob/Sub/ Non-surgical					
Radiation	6.503	0.039		4.578	0.101
No/Yes					
Chemotherapy	10.408	0.001		8.856	0.003
No/Yes					
ELN: examined lymph node; Sub: sublobar resection; Lob: lobectomy.					

**3:** Ia期SCLC患者总生存和肺癌特异性生存的多变量分析 Multivariate analysis of overall survival and lung cancer specific survival for Ia SCLC

Variables	Multivariate analysis				
	Overall survival			Lung cancer specific survival	
	Hazard ratio (95%CI)	*P*		Hazard ratio (95%CI)	*P*
Age (yr)		0.196			0.203
60-74	1.00 (reference)			1.00 (reference)	
75	1.155 (0.928-1.437)			1.164 (0.921-1.472)	
Gender		0.229			0.476
Male	1.00 (reference)			1.00 (reference)	
Female	0.885 (0.725 to 1.080)			0.925 (0.747-1.146)	
Race		0.173			0.393
White	1.00 (reference)			1.00 (reference)	
Non-white	1.221 (0.916-1.627)			1.145 (0.839 to 1.564)	
Location					
Upper	1.00 (reference)			1.00 (reference)	
Middle	1.385 (0.926-2.070)	0.113		1.229 (0.788-1.916)	0.363
Lower	1.164 (0.938-1.445)	0.168		1.266 (1.006-1.593)	0.044
Tumor size (cm)		0.860			0.076
≤2	1.00 (reference)			1.00 (reference)	
2-3	1.108 (0.831-1.247)			1.219 (0.980-1.516)	
ELN count		< 0.001			< 0.001
None	1.00 (reference)			1.00 (reference)	
≥1	0.516 (0.395-0.674)			0.564 (0.428-0.742)	
Surgical Procedure					
Non-surgical	1.00 (reference)			1.00 (reference)	
Sub	0.499 (0.351-0.708)	< 0.001		0.467 (0.318-0.683)	< 0.001
Lob	0.301 (0.222-0.407)	< 0.001		0.301 (0.218-0.414)	< 0.001
Radiation		< 0.001			< 0.001
No	1.00 (reference)			1.00 (reference)	
Yes	0.549 (0.435-0.693)			0.564 (0.439-0.724)	
Chemotherapy		0.018			0.021
No/unknown	1.00 (reference)			1.00 (reference)	
Yes	0.761 (0.607-0.953)			0.750 (0.588-0.957)	

## 3 讨论

众所周知，SCLC对放化疗敏感，但最终大多数会复发、其预后较其他类型肺癌差。本研究结果发现，对于年龄≥60岁T1N0M0的SCLC患者，肺叶切除的术后生存率明显高于亚肺叶切除（5年OS分别为44%和30%，*P*=0.028）。在总队列中，手术治疗队列相较于非手术队列能带来更好的预后（非手术队列5年OS仅为：14%）。此外，肺叶切除+辅助放化疗联合的患者生存率最高（5年OS为50%）。结果证实，肺叶切除联合术后放化疗治疗年龄≥60岁、肿瘤大小≤3 cm的SCLC患者的优势显著，而Ia期SCLC手术方案的选择上行亚肺叶切除并不能得到令人满意的结果，其预后较肺叶切除更差（中位OS分别为23个月和45个月，5年OS为30%和44%，*P*=0.028）。

Weksler等^[14]^的回顾性研究报道了3, 566例Ⅰ期或Ⅱ期SCLC患者，其结果显示手术患者的术后中位生存期（34个月）明显高于非手术患者（16个月，*P* < 0.001）；肺叶切除或全肺切除组术后的中位生存期为39个月，明显优于楔形切除组患者（28个月，*P*=0.001），该研究结果与本研究类似。我们认为，亚肺叶切除相较于肺叶切除治疗早期SCLC效果差，主要与SCLC恶性程度高、早期容易转移的特性相关；选择亚肺叶切除术式，虽然能更好地保护患者的肺功能，但可能对肿瘤所在肺叶内的潜在远处转移控制欠佳。针对SCLC侵袭强和早期易转移等特性，选择肺叶切除治疗切除范围更大，尽管较大程度损害了肺功能，但保证了病灶肺叶的完整切除，降低了潜在复发及转移的风险，对患者远期生存更有益。

本研究结果显示，亚肺叶切除+术后放化疗组相较于单纯肺叶切除组患者的生存预后，结果无统计学差异（*P*=0.759），故亚肺叶切除+辅助放化疗是否可改善生存还需进一步验证。有研究证实NSCLC患者行肺叶切除术+淋巴结采样/清扫检查，可改善患者的生存预后^[15]^，但本研究的SCLC手术队列中，行淋巴结采样/清扫检查与无淋巴结采样/清扫检查患者相比，预后差异无统计学意义（*P*=0.107）；但*Cox*多变量分析结果却显示，淋巴结检查是OS和LCSS有益的的独立预后因素（OS: HR=0.516, *P* < 0.001; LCSS: HR=0.564, *P* < 0.001）。造成单因素分析无统计学意义的原因，可能在于样本量少及潜在的选择性偏倚有关。

最新NCCN指南推荐Ⅰ期SCLC的治疗方式为手术+辅助化疗^[6]^，但SCLC选择手术治疗的现状却不尽人意。Wakeam等^[16]^研究纳入的9, 740例T1-2期SCLC患者的手术率仅为（23%），其中2/3可手术患者（即符合手术条件患者）未选择手术治疗。这与本研究纳入的Ia期老年SCLC患者的手术治疗率（32%）类似。因此，早期SCLC患者手术治疗率还有较大提升空间。Naidoo等^[17]^综述道，胸外科的VATS手术正在高速发展，新技术对比传统开胸手术有更多优点：术后疼痛减轻、住院时间缩短、气体漏出减少、肺炎和房性心律失常降低，同时炎症介质的释放也较开胸手术少，且VATS较于传统开胸手术的无病生存率和整体生存率是相当的。微创胸外科的发展，增加了患者手术机会，手术可选择的术式也随之增加。所以，我们相信未来对于早期SCLC的手术治疗及术式的选择会有更多的关注与改进。

本研究中，T1N0M0 SCLC（≤3 cm）在接受肺叶切除术+辅助放化疗的患者5年OS最高（50%），这说明，即使Ia期SCLC，辅助放化疗对其预后也至关重要。这与NSCLC不同，Liang等^[18]^在其综述中总结道：NSCLC在Ia期术后进行化疗对于生存率无明显改善，Ib期是否行辅助化疗仍有争议，故一般在可切除的Ia期NSCLC患者术后不推荐行辅助化疗，而辅助放疗在NSCLC中的价值有待证实^[19]^。邵为朋等^[20]^在对SCLC治疗策略的综述中提到：目前针对SCLC的治疗仍然是延续近30年传统的放化疗为基础的治疗，并展望未来多方式综合治疗（放化疗+靶向治疗+免疫治疗）或许能给SCLC患者带来一线生机。因此，不管Ia期SCLC切除术式如何选择，其术后放化疗的效果也不可忽视。我们期待未来在手术治疗SCLC的基础上，搭配放化疗+靶向治疗+免疫治疗等综合治疗能给患者带来更好的治疗效果。

然而，本研究仍存在以下局限。第一，回顾性分析的研究性质；第二，虽然本研究纳入了SEER数据库共515例Ia期SCLC患者，选择亚肺叶切除治疗的这部分早期患者仍存在一定的选择性偏倚；且可能造成此偏倚患者的肺功能、伴随的心血管疾病等信息在SEER数据库中无法获得。第三，本研究纳入的Ia期SCLC患者病例数量仍偏少、术后随访时间较短。

总之，本研究通过对比肺叶切除和亚肺叶切除治疗Ia期SCLC的预后分析，结果证实Ia期患者行亚肺叶切除治疗预后较肺叶切除差。针对年龄≥60岁的T1N0M0（≤3 cm）可手术切除SCLC患者，我们建议首选解剖性肺叶切除+辅助放化疗治疗。在新技术快速发展及其带来许多优势前提下，本研究结果的科学性和普遍性还需将来多中心、大样本及前瞻性的临床研究来进一步证实和完善。
